# Fatal Case of Pneumonia Associated with Pandemic (H1N1) 2009 in HIV-Positive Patient

**DOI:** 10.3201/eid1601.090930

**Published:** 2010-01

**Authors:** Natalie C. Klein, Azfar Chak, Marilyn Chengot, Diane H. Johnson, Burke A. Cunha

**Affiliations:** Winthrop-University Hospital, Mineola, New York, USA; State University of New York School of Medicine, Stony Brook, New York, USA

**Keywords:** Pandemic (H1N1) 2009, influenza, pneumonia, HIV, viruses, expedited, letter

**To the Editor:** Pandemic (H1N1) 2009 virus first appeared in March 2009 in Mexico. In June 2009, a pandemic was declared by the World Health Organization (*1*). Influenza A virus (H1N1) caused a pandemic in 1918–1919; estimated deaths were ≈100 million worldwide (*2*). Symptoms of pandemic (H1N1) 2009 are similar to those of seasonal influenza (fever, cough, sore throat, body aches, headache, chills, and fatigue) (*3*). Pandemic (H1N1) 2009 should be considered in the differential diagnosis of patients with acute febrile respiratory illness who have been in contact with persons with confirmed influenza or reside in areas where influenza has been reported (*2*).

Although most cases of pandemic (H1N1) 2009 in the United States have been mild, 2%–5% of infected persons have required hospitalization (*2*). Immunosuppressed persons, the elderly, persons with underlying lung or cardiac disease, pregnant women, persons with diabetes, obese persons, and children <5 years of age are at increased risk for this disease (*4*).

We report pneumonia associated with pandemic (H1N1) 2009, which resulted in respiratory and renal failure and death, in a 39-year-old HIV-positive woman. She had type 1 diabetes and a diagnosis of AIDS 7 years ago and had received highly active antiretroviral therapy. She also had an ill child at home with an influenza-like illness.

Her medical history included pleuropericardial *Nocardia* spp. infection, recurrent pleural effusions requiring thoracentesis, and hepatomegaly of unknown cause. Her most recent CD4 cell count was 166 cells/μL with undetectable viral load 1 month before admission. Medications prescribed included combivir, efavirenz, and trimethoprim/sulfamethoxazole but she was noncompliant. She had received the 2008–09 seasonal influenza vaccine and pneumococcal vaccine.

The patient was admitted to Winthrop-University Hospital (Mineola, NY, USA) on June 5, 2009, for community-acquired pneumonia. She received empiric moxifloxacin and atovaquone. Because of concern for persistent *Nocardia* spp. infection, she was also treated with doxycycline. The result of a rapid influenza test (QuickVue; Quidel, San Diego, CA, USA) was negative for a nasal swab specimen on day 1 of hospitalization. Over the next 48 hours, her clinical status deteriorated, and she experienced worsening hypotension and respiratory distress.

On admission, she had fever (101°F) for 3 days, pulse rate of 109 beats/min, blood pressure of 86/52 mm Hg, respiratory rate of 22 breaths/min (oxygen saturation of 88% on room air), generalized weakness, nonproductive cough, and increasing shortness of breath. She was alert and oriented. Physical examination showed decreased breath sounds at bases, hepatomegaly, and bilateral edema in the lower extremities. Laboratory tests showed 3,000 leukocytes/mm^3^ (93% neutrophils, 2% bands, and 3% lymphocytes), hemoglobin level of 12.7 g/dL, and 118,000 platelets/mm^3^. Other laboratory values were blood urea nitrogen 66 mg/dL, creatinine 2.9 mg/dL, creatinine phosphokinase 2,276 IU/L, and lactic acid 3.6 mmol/L (anion gap 13). A chest radiograph showed moderate pleural effusion in the right lung and retrocardiac air space disease. Test results for influenza virus, respiratory fluorescent antibodies (D3 Ultra DFA Respiratory Virus Screening and Infectious Disease Kit; Diagnostic Hybrids, Inc., Athens, OH, USA), and virus culture were negative.

The patient was transferred to the intensive care unit and required intubation, pressor support, and continuous venovenous hemofiltration for fluid removal. Empiric oseltamivir (150 mg 2×/d) was started on hospital day 3; moxifloxicin was discontinued, and meropenem was given for pneumonia (*5*). Thoracentesis showed transudative fluid negative for acid-fast bacilli, bacteria, and fungi.

Results of blood cultures and urine analysis for *Legionella* spp. antigen were negative. Repeat chest radiography showed a right-sided pneumothorax and worsening bilateral airspace disease. A chest tube was inserted in the right lung, and bronchoscopy was performed on hospital day 5. Results of bronchoalveolar lavage were negative for *Pneumocystis jiroveci*, virus inclusions, fungi, acid-fast bacilli, bacteria, and mycobacteria. However, clusters of filamentous organisms were seen. On hospital day 5, results of a second rapid influenza test, respiratory fluorescent antibody test, and nasopharyngeal virus culture were negative. Diagnosis was based on a positive result for pandemic (H1N1) 2009 by real-time reverse transcription–PCR (RT-PCR) for a nasopharyngeal swab specimen (New York State Department of Health). Despite empiric treatment with oseltamivir, the patient died on June 15, 2009 (day 11 of hospitalization).

Symptoms of pandemic (H1N1) 2009 in HIV-infected persons are not known. However, these persons have a higher risk for complications. In previous seasonal influenza outbreaks, HIV-infected persons had more severe infections and increased hospitalization and mortality rates (*6*).

Although a diagnosis of pandemic (H1N1) 2009 was first considered for our patient because of her ill child, she was not initially treated with oseltamivir because of the negative influenza test result and concern for opportunistic infections. Only the result of an RT-PCR for pandemic (H1N1) 2009 was positive. No other pathogens were detected in her blood, urine, sputum, bronchoalveolar lavage, or thoracenthesis fluid.

Empiric treatment in patients with pandemic (H1N1) 2009 should be considered in those seeking treatment for influenza-like symptoms, especially in the setting of sick contacts with respiratory illnesses. Rapid influenza tests, respiratory fluorescent antibody tests, and viral cultures may not provide a diagnosis. An RT-PCR for pandemic (H1N1) 2009 may be needed to provide a diagnosis.
